# Advancing Vaccinology Capacity: Education and Efforts in Vaccine Development and Manufacturing across Africa

**DOI:** 10.3390/vaccines12070741

**Published:** 2024-07-03

**Authors:** Jean Paul Sinumvayo, Pierre Celestin Munezero, Adegboyega Taofeek Tope, Rasheed Omotayo Adeyemo, Muritala Issa Bale, Jean Baptiste Nyandwi, Vetjaera Mekupi Haakuria, Leon Mutesa, Ahmed Adebowale Adedeji

**Affiliations:** 1 Department of Microbiology and Parasitology, School of Medicine and Pharmacy, College of Medicine and Health Sciences, University of Rwanda, Huye P.O. Box 117, Rwanda; munezeropierrecelestin@gmail.com (P.C.M.); t.adegboyega@ur.ac.rw (A.T.T.); yemodek2000@yahoo.com (R.O.A.); muribaale@gmail.com (M.I.B.); 2 East African Community, Regional Center of Excellence for Vaccines, Immunization and Health Supply Chain Management (EAC RCE-VIHSCM), Kigali P.O. Box 3286, Rwanda; nbaptiste1988@gmail.com (J.B.N.); haakuria@gmail.com (V.M.H.); 3 Future of Medicine, Science, Technology and Innovation Research Group, School of Medicine and Pharmacy, University of Rwanda, Rwanda, Kigali P.O. Box 3286, Rwanda; ahmedade1@yahoo.co.uk; 4 Department of Pharmacology and Toxicology, School of Medicine and Pharmacy, College of Medicine and Health Sciences, University of Rwanda, Huye P.O. Box 117, Rwanda; 5 Department of Biochemistry, Molecular Biology and Genetics, School of Medicine and Pharmacy, College of Medicine and Health Sciences, University of Rwanda, Huye P.O. Box 117, Rwanda; lmutesa@gmail.com; 6 Center for Human Genetics, College of Medicine and Health Sciences, University of Rwanda, Kigali P.O. Box 4285, Rwanda

**Keywords:** vaccinology, African countries, education, vaccine manufacturing

## Abstract

Africa, home to the world’s second-largest population of approximately 1.3 billion, grapples with significant challenges in meeting its medical needs, particularly in accessing quality healthcare services and products. The continent faces a continuous onslaught of emerging infectious diseases, exacerbating the strain on its already fragile public health infrastructure. The COVID-19 crisis highlighted the urgency to build local vaccine production capacity and strengthen the health infrastructure in general. The risks associated with a heavy reliance on imported vaccines were exposed during the COVID-19 pandemic, necessitating the need to nurture and strengthen the local manufacturing of vaccines and therapeutic biologics. Various initiatives addressing training, manufacturing, and regulatory affairs are underway, and these require increasing dedicated and purposeful financial investment. Building vaccine manufacturing capacity requires substantial investment in training and infrastructure. This manuscript examines the current state of education in vaccinology and related sciences in Africa. It also provides an overview of the continent’s efforts to address educational needs in vaccine development and manufacturing. Additionally, it evaluates the initiatives aimed at strengthening vaccine education and literacy, highlighting successful approaches and ongoing challenges. By assessing the progress made and identifying the remaining obstacles, this review offers insights into how Africa can enhance its vaccine manufacturing capacity to respond to vaccine-preventable disease challenges.

## 1. Introduction

Africa grapples with the formidable challenge of insufficient access to medical products and services, notably vaccines and therapeutics. The recent surge in demand for vaccines across nearly all African nations in the wake of the Coronavirus disease of 2019 (COVID-19) pandemic serves as a stark reminder of the continent’s pressing need to address this issue. Despite the implementation of expanded immunization programs in the early 1970s, Africa’s endeavors to mitigate vaccine-preventable diseases remain far from satisfactory [1]. The challenge of accessing vaccines and vaccine-related products persists due to a lack of local manufacturing capacity. This issue is exacerbated by the critical need for timely access to essential vaccines, particularly during pandemic outbreaks such as the recent emergence of severe acute respiratory syndrome coronavirus 2 (SARS-CoV-2). Presently, Southern Africa, encompassing nations such as Zambia, Zimbabwe, Malawi, and Mozambique, is confronting a cholera outbreak that is further complicated by a shortage of the cholera vaccine [2,3]. The relentless surge of emerging infectious diseases with regional or global implications has placed immense strain on the quality of healthcare in Africa. The continent’s weak and fragmented public health infrastructure exacerbates this pressure, creating significant challenges in effectively responding to these diseases and safeguarding public health [4].

The affordability of healthcare and medical products including vaccines and therapeutics poses a significant barrier for many in sub-Saharan Africa, where poverty levels are high. Consequently, both cost and supply constraints further hinder access to essential medical services and healthcare products. These challenges are compounded by inadequate public health facilities, encompassing deficiencies in physical infrastructure, human resources, and medical supplies. Moreover, the increased prevalence of infectious diseases exacerbates the strain on an already overstretched healthcare system [5,6].

Despite efforts to improve healthcare infrastructure, sub-Saharan Africa continues to grapple with limited health facilities, poorly equipped health systems, and challenges in pharmaceutical supply chains. Consequently, the burden of disease in the region persists. Given these challenges, vaccination remains the most prudent approach to mitigating the impact of high disease burden in sub-Saharan Africa.

Limited focus on vaccine development and production in certain regions has hampered global efforts to achieve widespread vaccination coverage, posing a significant bottleneck in controlling the spread of contagious diseases. Collaborative initiatives involving national governments, the WHO, and allied partners are crucial to address this issue. Insufficient attention to vaccine development undermines efforts to limit disease transmission, exacerbating associated morbidities and mortalities, thus necessitating prioritized investment in robust vaccine infrastructure for resilient global health responses [7,8,9].

The recent COVID-19 outbreak, coupled with the region’s weak public health infrastructure and challenges in accessing essential COVID-19 vaccines through initiatives like the COVAX facility, has underscored the urgent need to develop local vaccine manufacturing capacity.

Research findings underscore Africa’s heavy reliance on imported vaccines, with 99% of its supply originating from international sources [10,11]. Despite being the recipient of approximately 25% of global vaccine production [12,13], the continent faces a stark reality where the vast majority of vaccines consumed within its borders are imported rather than domestically produced. This reliance on imported vaccines highlights the urgent need for Africa to bolster its vaccine manufacturing capabilities to achieve health security and self-sufficiency. Various initiatives including training programs targeting manufacturing, diagnostics, regulatory affairs, and surveillance are underway to address this pressing need [14,15]. However, inadequate investment levels in this sector underscore the necessity for broader and more substantial financial commitments to effectively enhance Africa’s vaccine manufacturing capacity [14,15].

The objectives of this review are to identify gaps in knowledge and capacity, propose solutions to strengthen education, and build capacity in vaccine development and manufacturing on the continent. Additionally, the current review aims to provide a comprehensive situational analysis of the vaccine manufacturing landscape on the continent and to examine the various efforts and initiatives to enhance vaccine manufacturing capacity in Africa.

## 2. Methodology

The authors relied on a comprehensive array of published academic resources to inform this review. These included databases such as PubMed, a National Institute of Health online database housing abstracts, as well as articles on biomedical sciences sourced from platforms like the National Center for Biotechnological Information, Google Scholar, and databases from reputable organizations like the World Health Organization (WHO) and the Centers for Disease Control and Prevention (CDC), among others.

To ensure a comprehensive exploration of the topic, the authors employed key search terms and phrases such as “vaccine”, “vaccinology”, “vaccine development”, “vaccination in Africa”, “vaccinology courses and training”, and “the future of vaccines in Africa”, among others. This search strategy targeted original research articles, systematic review papers, meta-analyses, research letters, organization documents, policies, and case reports closely related to the themes addressed in the current review.

## 3. The African Vaccinology Landscape

The African continent faces significant challenges in providing healthcare to its population, particularly concerning access to vaccines and related products. A notable hurdle is the limited local manufacturing capacity for vaccines, exacerbating the difficulty in ensuring widespread access, particularly during outbreaks like the ongoing SARS-CoV-2 pandemic. Currently, Southern Africa, encompassing nations such as Zambia, Zimbabwe, Malawi, and Mozambique, is contending with a cholera outbreak that is further complicated by a shortage of cholera vaccines [2,3].

Moreover, yellow fever outbreaks have surged across West Africa to Central Africa in 2024, as reported by the WHO [16]. Additionally, the Africa CDC has documented 5427 combined cases of dengue fever in Ethiopia, Mali, and Mauritius in its Weekly Event-Based Surveillance Report for March 2024. These diseases add to the longstanding threats of Ebola virus disease (EVD) and malaria, which continue to burden the continent’s healthcare systems [17,18]. Despite these challenges, there have been some promising advancements. The introduction of the new Ebola vaccine, V920/ERVEBO or rVSV-ZEBOV, has shown remarkable efficacy in reducing infections and mortality rates by up to 50%. Furthermore, the development of Matrix-M (R-21), exhibiting 75% efficacy against the prevalent strain of malaria, presents a significant breakthrough in combating this debilitating disease [18]. However, the relentless emergence of infectious diseases with potentially catastrophic regional or global implications continues to strain Africa’s already fragile and fragmented public health infrastructure. Addressing these challenges demands concerted efforts in bolstering healthcare systems, enhancing vaccine access and distribution networks, and fostering collaboration among nations and international organizations to mitigate the impact of infectious diseases on the continent’s population [4].

## 4. State of Vaccinology Training in Africa

The global landscape of vaccinology faces formidable challenges, notably in vaccine development and manufacturing. In Africa, achieving widespread immunity hinges on vaccinating a substantial portion of the populace, and is impeded by the lack of local vaccine production capacity due to lack of technical expertise, the cost involved in setting up manufacturing plants, and supply limitations. Moreover, bolstering capacity in vaccine development necessitates strategic investments in training and research infrastructure at academic and research institutions. Additionally, facilitating translational research requires equipping researchers with entrepreneurship skills to bridge the gap between academia and industry to facilitate the translation of research discoveries into practical solutions. This entails providing a supportive ecosystem for taking spin-outs from the bench to market.

### 4.1. Vaccinology Courses

Across the globe, a diverse array of vaccinology courses cater to varying levels of expertise, often categorized as advanced programs. These courses serve as vital platforms for knowledge dissemination and skills enhancement. Some offerings focus on short-term training initiatives tailored to decision-makers in governmental and non-governmental sectors, industry professionals, and academia. These programs aim to equip participants with comprehensive backgrounds in diverse aspects of vaccinology. Topics covered typically include vaccine development, vaccine manufacturing unit operations, facility design, bioprocess development regulatory procedures, clinical trial methodologies, vaccine-specific concerns, and fundamental immunology principles, among other pertinent subjects. By providing sustainable education and training opportunities, these courses contribute significantly to advancing global efforts in vaccine research, development, and deployment [19,20].

Introducing postgraduate diploma or certificate programs as well as master degree courses ranging from one to two years in duration could significantly enhance the productivity of the vaccinology workforce, particularly in regions such as Africa, where there is a pressing need for skilled professionals in this field. In 2018, a total of 33 vaccine-related courses were offered globally, comprising seventeen short-term courses, eleven postgraduate programs, and five master degree training initiatives [21]. As an example, the 23rd Advanced Course of Vaccinology (ADVAC) stands as a testament to the value of such programs in strengthening leadership skills in vaccinology (https://www.fondation-merieux.org/en/events/23rd-advanced-course-of-vaccinology-advac/ (accessed on 5 May 2023)).

In preparation for the inaugural global vaccinology training workshop in 2018, a survey was conducted to evaluate the state of vaccinology education worldwide. The findings revealed that nearly half of the vaccinology courses faced significant challenges due to insufficient funding and concerns regarding sustainability. Moreover, only a limited number of courses prioritized funding opportunities for participants from low- and middle-income countries (LMICs) [20]. Duclos et al. (2019) [19] discussed various aspects of the vaccinology landscape in their report from the Advanced Vaccine Courses workshop. They highlighted critical areas such as technology transfer, vaccine development, and program operations, underscoring the shortage of well-trained scientists both within and originating from LMICs [19]. In addition to these courses, the Kina Foundation, a non-profit organization in collaboration with the Noguchi Memorial Institute for Medical Research, College of Health Sciences, University of Ghana, is offering a 4-month certificate course in Vaccine Biomanufacturing [22]. This course is designed to equip participants with practical hands-on skills in the industrial vaccine biomanufacturing process, preparing them for work environments in vaccine biomanufacturing facilities across Ghana and the African continent. The curriculum also includes subject areas relevant to the biomanufacturing process, broadening the participants’ knowledge base. Overall, short courses in vaccinology and vaccine development and manufacturing appear to be acutely inadequate on the continent.

### 4.2. Current State of Education in Vaccinology in Africa

The continuous emergence of infectious diseases in Africa and the advent of the COVID-19 pandemic have prompted stakeholders to reassess vaccination-related issues across the continent. One outcome of this focus is the global vaccine action plan, which is aimed at achieving equitable access to vaccines by 2020, and underscores the importance of providing vaccination to people worldwide within a 10-year vision [23].

Since the advent of the SARS-CoV-2 pandemic, various training centers have been established to advance the global vaccine action plan’s objectives. One notable institution among these is the East Africa Center for Vaccines and Immunization (ECAVI), located at Egerton University in Kenya. ECAVI’s inception aimed to mitigate morbidity and mortality linked to malignancies and vaccine-preventable diseases within East African nations. Through its multifaceted approach encompassing advocacy, research, and education, ECAVI endeavors to strengthen vaccination health systems. Additionally, it focuses on fostering acceptance and providing training on novel and accessible vaccines [24]. In the same context, the African Vaccine Regulatory Forum plays a pivotal role in supporting member states across African countries by providing training in good clinical practices and clinical trial oversight, thereby strengthening their technical capacity [25]. This commitment to capacity-building reflects a strategic response to the evolving landscape of vaccination initiatives, which now extend beyond traditional demographics to include the elderly [21]. This expansion is informed by the lessons learned from the COVID-19 pandemic, highlighting the necessity of preparing for future health crises including potential biological threats [26].

Furthermore, the Vaccine for Africa Initiative, established in 2005, has been instrumental in addressing the escalating demand for vaccinology training on the continent. By 2020, the initiative had successfully trained 958 individuals from 44 African countries, covering a diverse range of professionals, from scientists to policymakers, across Anglophone nations [27]. However, this success also brings to light concerns regarding language discrimination and limited access due to stringent selection criteria and funding constraints. To address these challenges, there is a growing recognition of the need for inclusive training programs that cater to linguistic diversity and provide opportunities for broader participation. Additionally, the emergence of e-vaccinology courses and translation services offers promising avenues for promoting inclusivity and expanding the reach of vaccinology education initiatives [27]. Other institutions have also emerged to meet the growing need for expertise in vaccinology. One notable example is the Jenner Institute at the University of Oxford in the United Kingdom. This institute offers postgraduate-level courses specifically tailored to residents in Africa, focusing on various facets of vaccine development, manufacturing, ethical considerations, and approval processes. These courses aim to equip individuals with comprehensive knowledge and skills essential for advancing vaccinology in Africa and effectively addressing public health challenges. By providing such training opportunities, institutions like the Jenner Institute play a crucial role in building the human capacity necessary to drive innovation and progress in the field of vaccinology on the African continent [28].

Furthermore, in a study conducted by Moïsi et al. (2019) [29], 10 vaccinology courses providing training across various themes were identified in 2019. Among these courses, seven were categorized as short courses, with five aimed at training health professionals or immunization managers on fundamental concepts of vaccinology and program implementation issues. Additionally, two advanced short courses focused specifically on vaccine design, development, and policy for scientists. Furthermore, the study identified three long-term training programs, spanning nine to eighteen months, categorized as Master’s programs. These Master’s programs had a distinct focus on biomedical and management fields. The diverse range of training offerings, encompassing short courses for practical skill development and Master’s programs for in-depth theoretical understanding, highlights the multifaceted nature of vaccinology education. This diversity underscores the importance of building expertise across various sectors of the healthcare industry to effectively address the complex challenges associated with vaccination initiatives [29].

The University of Rwanda’s hosting of the East Africa Community (EAC) Regional Center of Excellence for Vaccines, Immunization, and Health Supply Chain Management (RCE-VIHSCM) underscores a steadfast commitment to combatting vaccine-preventable diseases and overcoming logistical hurdles within the region. Established during the 9th Ordinary Meeting of the EAC Sectoral Council of Ministers of Health on 17 April 2014, in the United Republic of Tanzania, this initiative symbolizes a unified effort among East African nations to strengthen their capacity in critical areas of healthcare delivery [30]. Aligned with broader objectives to modernize medical services and incorporate electronic health and information communications technology across the EACs, the center offers a diverse array of training programs. These programs, ranging from short-term courses focused on enhancing the Extended Program on Immunization to long-term courses targeting national healthcare systems, play a pivotal role in bridging gaps and bolstering healthcare infrastructure throughout the EAC Partner states. Furthermore, in line with its commitment to addressing various facets of healthcare and pharmaceutical sciences, the center has expanded its academic offerings to include additional Master’s programs. These new programs such as the MSc in Vaccinology, MSc in Pharmaceutical Analysis and Quality Assurance, and MSc in Regulatory Affairs complement the existing Master’s degree course in Health Supply Chain Management. This diversification of academic offerings underscores the center’s dedication to catering to the evolving needs of healthcare professionals and researchers in the region [31]. Furthermore, the proliferation of vaccinology-related courses across Africa, spanning regions such as South, West, and North Africa, underscores a continent-wide dedication to strengthening expertise and comprehensively addressing public health challenges. These tailored training initiatives hold promise in equipping healthcare professionals with the necessary knowledge and skills to effectively combat vaccine-preventable diseases and improve health outcomes for populations across Africa. Through collaboration and knowledge exchange, these efforts contribute to sustainable progress toward achieving universal healthcare and reducing the burden of preventable illnesses across the continent [32,33].

The significance of vaccinology training for African citizens within their respective countries does not diminish the value of similar training undertaken by Africans in the Global North. Programs like the ADVAC and various degree programs in vaccinology offered in regions such as Europe and North America play a pivotal role in augmenting the pool of trained African professionals and enhancing their expertise in the field.

By participating in these programs, African professionals gain access to cutting-edge research, advanced methodologies, and international networks, which enrich their experience and broaden their perspectives in vaccinology. Moreover, collaboration with institutions in the Global North fosters knowledge exchange and facilitates the transfer of best practices, ultimately contributing to the advancement of vaccination initiatives and public health outcomes across Africa.

While advancements in vaccinology education and training have been notable, challenges persist in developing vaccines for emerging and re-emerging infectious diseases like Ebola. Despite the existence of licensed Ebola vaccines such as the single-dose Ad5-EBOV from China and the 2-dose rVSV/Ad5 from the Russian Federation, there remain investigational vaccines like rVSV-ZEBOV from Merck and Ad26, ZEBOV/MVA-BN-Filo from Janssen. Understanding the causes of delays in vaccine development is crucial, as it often involves addressing gaps in research, funding, infrastructure, and regulatory processes. By overcoming these barriers, the global community can accelerate progress toward developing effective vaccines for emerging infectious diseases. This concerted effort is vital for saving lives and safeguarding public health not only in Africa, but also worldwide [34,35,36]. It is possible that the African experience in addressing vaccine-related challenges may still be influenced by the priorities and perspectives of European and American entities, which may not always align perfectly with the needs of the African continent. While there is recognition that education and training programs in vaccinology are currently limited compared to the demands of the continent, efforts are underway to address this gap and increase the number of vaccinologists in Africa. It is incumbent on Africa to build the requisite expertise and infrastructure to promote the development of essential vaccines for its population.

Various programs have been initiated across different parts of Africa to provide training in vaccinology and related sciences at the master’s level and through advanced training courses. These initiatives aim to equip individuals with the necessary knowledge and skills to address the unique challenges faced by African countries in the field of vaccinology. By expanding access to high-quality education and training opportunities, Africa can build a strong cadre of vaccinology experts capable of driving innovative solutions and advancements in public health across the continent (Table 1).

In this regard, the Southern region of Africa ranks first with a proportion of 120% of programs in vaccinology and related sciences in five countries (Table S1). The Northern region (Table S2) ranks second with a proportion of 71.4% of programs in seven countries, followed by the Western region (62.5% in 16 countries) (Table S3). The Central African region ranks fourth with 55.6% of programs in nine countries (Table S4), while the Eastern region ranks fifth in terms of vaccinology programs with a proportion of 26.3% in 19 countries (Table S5). These proportions show the progress made so far, however, the unequal distribution of these training courses across countries calls for additional effort. Only 21 countries possess at least one training program in vaccinology, and 64.3% of African countries need to move to others countries for any kind of training in vaccinology. In East Africa, around 80% of countries cannot locally manage to offer vaccinology training. A limited number of training programs in vaccinology and vaccine-related courses may result in a shortage of skilled professionals in the field within African countries. This scarcity of expertise could, in turn, impact the capacity of African nations to independently engage in vaccine research, development, and production. Without a robust workforce trained in vaccinology, African countries may face challenges in conducting essential research, navigating regulatory processes, and establishing the infrastructure necessary for vaccine production. Consequently, the reliance on external expertise and resources from other regions may hinder the autonomy and decision-making capacity of African countries in the development and production of vaccines tailored to their specific health needs and priorities. Thus, the availability of access to comprehensive training programs in vaccinology are crucial factors that can influence the ability of African nations to pursue self-sufficiency in vaccine production and effectively address public health challenges.

## 5. The Technology Platforms

The challenges in accessing COVID-19 vaccines have highlighted not just the need for capacity building and training, but also the importance of adopting new vaccine production technologies like mRNA vaccines. Despite initial skepticism, mRNA vaccines have proven effective against COVID-19, opening doors for their potential use against other diseases like human immunodeficiency virus (HIV), tuberculosis (TB), and malaria. However, widespread adoption faces logistical and public perception challenges. Continued investment in research and training is essential to optimize mRNA vaccine technology and ensure equitable access to these lifesaving tools [37].

The mRNA vaccine technology involves selecting and engineering the target open reading frames (ORFs) that encode the desired protein and formulating it into a vaccine. This process lies at the heart of mRNA vaccine technology, enabling the production of specific proteins in vivo that trigger an immune response in the host body. By carefully selecting and modifying these ORFs, researchers can tailor mRNA vaccines to target a wide range of pathogens, offering immense potential for combating infectious diseases and beyond [38]. The selection and engineering of target ORFs in mRNA vaccine technology require extensive knowledge of microbial biology, pathogenesis, host–microbe interaction, and bioinformatics. This process is crucial for customizing gene synthesis to produce specific proteins that trigger an immune response. Additionally, while certain vaccine components can be sourced from various companies, comprehensive education and training programs are essential to address the challenges associated with mRNA technology. These programs should focus on vaccine design, development, and manufacturing, particularly targeting second- and third-generation mRNA vaccines.

## 6. The WHO mRNA Technology Transfer Program

The power of mRNA technology has been demonstrated in the higher effectiveness (VE) and development in a shorter time period. The WHO, in partnership with Medicine Patent Pool (MPP), have established a training program structured in a hub and spoke model to avail training in mRNA technology and subsequent technology transfer to LMICs. Through this program, 15 countries have been trained and technology transfer completed to enable rapid response to future pandemic outbreaks.

To realize a robust local capacity in mRNA vaccine-related technologies, several strategic initiatives are essential. First, there needs to be a widespread deployment of scientists within mRNA-based biopharmaceutical companies. This deployment not only facilitates hands-on experience, but also encourages knowledge sharing and collaboration within the field. However, it is crucial to recognize that simply deploying scientists is not enough, there must also be a concerted effort to ensure they receive the necessary training and support.

Encouraging African governments to provide scholarships to young scientists focusing on mRNA vaccine technologies is a pivotal step. Scholarships can incentivize students to pursue careers in this field, ensuring a steady influx of talent. Additionally, universities with existing research groups in mRNA technology should play a pivotal role in training the next generation of scientists. These institutions can offer specialized programs and practical training opportunities, equipping students with the skills needed to drive innovation in vaccine development. A critical issue in this regard is to retain such technical talent to stem the brain drain. This requires investment in research and development facilities to gainfully employ this highly technical workforce.

The mention of the Master’s degree training program in Biotechnology launched in Rwanda is particularly noteworthy. This initiative exemplifies the proactive approach needed to address the skills gap in Africa. By offering specialized training programs, countries like Rwanda can nurture a pipeline of skilled professionals poised to contribute to vaccine research and development. Another promising example is the efforts of RCE-VIHSCM in initiating programs such as the MSc in Vaccinology. This initiative aims to train more scientists in this crucial field, paving the way for a future with a greater pool of trained professionals dedicated to advancing vaccine science. Furthermore, collaboration with international researchers is essential for knowledge exchange and capacity building. African scientists must have opportunities to engage with global experts, participate in collaborative projects, and leverage international resources and expertise. This collaborative approach not only accelerates progress, but also fosters a culture of innovation and excellence within Africa’s scientific community.

However, economic challenges loom large. The costs associated with training scientists, acquiring raw materials, conducting clinical trials, and establishing vaccine manufacturing facilities are significant. To overcome these challenges, partnerships between governments, academia, industry, and international organizations are essential. These partnerships can mobilize resources, share costs, and streamline processes, ultimately making vaccine development more accessible and sustainable in Africa [39,40]. To achieve this, African governments must strike a balance between investing in vaccine production for prevention, prioritizing diseases that pose the greatest threat to the continent, ensuring the affordability of treatments and vaccines, and investing in education and capacity building.

## 7. Current Efforts of Africa in Vaccine Manufacturing and Future Perspectives

Numerous newly emerging and re-emerging infectious diseases impose a significant public health burden not only in Africa, but also globally. Regrettably, many of these diseases lack effective vaccines, exacerbating the challenges faced in combating them. Among the array of diseases without vaccines are human African trypanosomiasis, HIV, plague, hepatitis C, drug-resistant malaria, Rift Valley fever, Zika viruses, Lassa fever, Marburg or hemorrhagic fever, human monkeypox, non-Zaire strains of EVDs, extensively drug-resistant TB, multidrug-resistant TB, cholera, respiratory syncytial virus, and the recently emerged COVID-19 pandemic [41,42]. The significance of addressing these gaps is underscored by Table 2, which provides an overview of global efforts aimed at combatting emerging and re-emerging infectious diseases.

## 8. Assessment of Current Vaccine Production Capacity in Africa

The past three years have been particularly challenging due to the harsh health, economic, and social impacts of the COVID-19 crisis. This global pandemic prompted developing countries, especially in Africa, to reevaluate their health systems. Despite historical under-investment in the health sector, Africa responded urgently, leveraging collaborative efforts from governments, non-governmental organizations, private sectors, and scientific research institutions to establish and reform robust health institutions aligned with 21st-century science and technology.

With the advent of COVID-19, African leadership intensified their efforts to collaborate globally in developing effective and safe vaccines on the continent [94]. Notably, around ten vaccine manufacturers have emerged on the continent, primarily engaged in ‘fill and finish’ processes for imported products, with South Africa and Egypt leading in manufacturing COVID-19 vaccines [95,96]. In Senegal, for example, the *Institut Pasteur de Dakar* (IPD) manufactures yellow fever vaccines, while other countries such as Nigeria, Ethiopia, Kenya, Botwana, Cameroon, Chad, Ghana, Madagascar, Malawi, Mozambique, Niger, South Africa, Sudan, Tanzania, and Zimbabwe have also been manufacturing animal vaccines over the years and aspire to manufacture human vaccines [97].

For instance, Nigeria itself can produce vaccines such as the anthrax spore vaccine, Black Quarter vaccine, Brucella vaccine (S.19), contagious bovine pleuro-pneumonia vaccine, hemorrhagic septicemia vaccine, fowl cholera vaccine, fowl typhoid vaccine, and HantaVac [98]. Additionally, the National Veterinary Institute in Bishoftu, Ethiopia, produces 8 bacterial vaccines and 9 viral veterinary vaccines [99]. Cameroon produces a variety of vaccines including viral and bacterial vaccines as well as poultry vaccines [100]. Additionally, the Kenya Veterinary Vaccines Production Institute (KEVEVAPI) has been a longstanding producer of animal vaccines. KEVEVAPI has achieved significant milestones in vaccine production including vaccines for diseases such as turkey pox, Rift Valley fever, fowl pox, Newcastle disease, fowl typhoid, lumpy skin disease, foot and mouth disease, bluetongue disease, PPR disease, contagious caprine pleuropneumonia, sheep and goat pox, contagious bovine pleuropneumonia, and contagious pustular dermatitis [101].

This shows that the need for vaccine production is there and can inspire Africa to reach its 2060 agenda with enough vaccines. The reason for this is that the similarity in science between animal vaccines and human vaccines has a common point. Therefore, young scientists of Africa should learn these skills and then translate them for use in human vaccine design and production with improvement.

However, research indicates that Africa only produces 1% of its human vaccines and lacks adequate capacity to manufacture them at scale [10,11]. This means that a continent of 1.2 billion people still imports 99% of its vaccines, placing Africa at the top of the global vaccine demand [12,13].

## 9. Post COVID-19 Initiatives to Build Development and Manufacturing Capacity in Africa

Indeed, the Global Alliance for Vaccines Immunization (GAVI) has made significant efforts to introduce new vaccines in Africa [39]. However, concerns arise regarding the sustainability of such vaccines and whether countries would afford new vaccine introduction after graduation from GAVI [39]. Studies projecting Africa as the sole continent experiencing population and economic growth by the century’s end highlight its potential as a prime market for establishing local vaccine manufacturing hubs [39]. This trend underscores the importance of strategically investing in indigenous vaccine production capabilities across Africa. Doing so not only addresses the continent’s burgeoning healthcare needs, but also fosters economic development, bolsters health security, and reduces dependence on external sources for vaccines during emergencies. Ultimately, harnessing Africa’s growth for local vaccine manufacturing endeavors holds the promise of significant advancements in public health and socioeconomic progress.

The availability of robust animal vaccine production centers across the continent has motivated African leaders to remove all existing obstacles related to advancing local vaccine production including process development and maintenance, production facilities, life cycle management, and product portfolio management. Additionally, the rest of the world including United Nations (UN) agencies like the WHO and other private partners should continue supporting Africa to achieve high capacity building through postgraduate and postdoctoral training as well as advanced short courses.

Encouraging the establishment of well-equipped regional or national centers for vaccine research and development as well as pan-continental plants for the local production of research consumables is crucial. Furthermore, the prioritization of vaccine research funding by African governments and the establishment of a strong consortia of African research in vaccines for joint proposals and access to international research grants are of paramount importance.

The Africa CDC’s roadmap for vaccine manufacturing, targeting to produce 60% of the continent’s required vaccines by 2040, is acknowledged as a crucial step in bolstering health security and self-reliance. This initiative underscores the importance of sustained investment in local manufacturing capacity, technology transfer partnerships, regulatory harmonization, and infrastructure development, all of which are essential for achieving this ambitious goal [102].

New Partnership for Africa’s Development (NEPAD)‘s interventions, particularly the Pharmaceutical Manufacturing Plan for Africa (PMPA), are highlighted as instrumental in strengthening local pharmaceutical production capacity and reducing Africa’s dependence on imported medicines. Through strategic partnerships and targeted interventions, NEPAD contributes significantly to building a robust manufacturing ecosystem conducive to vaccine production.

The African Development Bank’s Industrialize Africa initiative is recognized for its focus on infrastructure development, skills training, and access to finance for manufacturing enterprises. By providing financial support for infrastructure projects and initiatives in skills development, the Africa Development Bank plays a crucial role in equipping African workers with the expertise needed for advanced manufacturing processes including vaccine production.

Furthermore, the role of regional economic communities (RECs) such as the Economic Community of West African States and EAC in fostering industrial cooperation and trade among member states is emphasized. These initiatives, which include harmonizing trade policies and promoting cross-border investment, create a conducive environment for manufacturing growth and contribute to the development of a vibrant manufacturing sector capable of producing vaccines and other essential goods.

Additionally, the policies and incentives of various national governments to attract investment in manufacturing are acknowledged for creating an enabling environment for industrial development. Examples such as tax incentives and sector-specific policies demonstrate the commitment of governments to enhancing Africa’s industrial landscape and fostering economic development.

**Figure 1:**
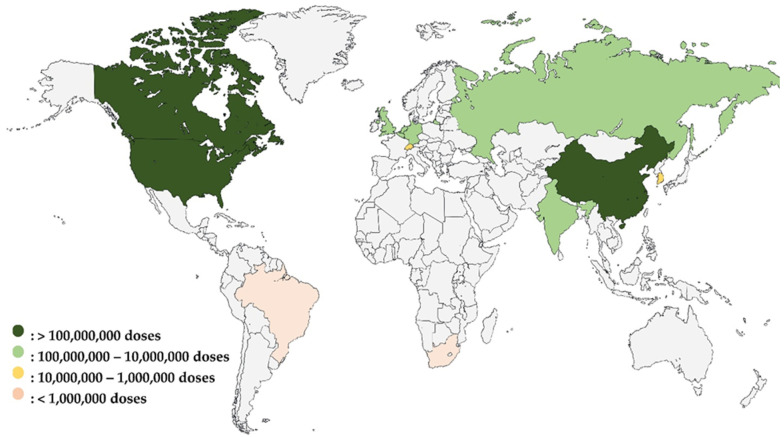
Global vaccine production capacity during the COVID-19 pandemic: pre-pandemic comparison and African representation [103].

As illustrated in Figure 1, the global vaccine production capacity during the COVID-19 pandemic highlighted significant disparities among countries. This diagram clearly shows that countries with existing vaccine manufacturing capacity were able to rapidly produce COVID-19 vaccines. In contrast, Africa, represented solely by South Africa’s Aspen Pharmacare, had limited capacity. To ramp up the production of doses during the pandemic, AstraZeneca outsourced the production of its vaccine to The Serum Institute of India while Aspen Pharmacare produced vaccines for Johnson & Johnson under a contract manufacturing agreement. In both cases, such contract manufacturing was based on the contract manufacturer having the existing capacity to produce vaccines. The figure also underscores the urgent need for Africa to build and enhance its vaccine manufacturing infrastructure to reduce its reliance on external sources and improve health security across the continent.

## 10. Enhancing Vaccine Manufacturing Capacity through Capacity Building, International Collaboration, and Investment in Africa

In response to the urgent challenges posed by the COVID-19 pandemic, Africa has embarked on a concerted effort to strengthen the Africa CDC and national public health institutions [11]. The continent’s strategy focuses on building local production capacities for vaccines, therapeutics, and diagnostics, reducing reliance on external sources. Efforts include attracting investments in public health workforce programs, fostering respectful partnerships, and providing comprehensive training in vaccine manufacturing to local scientists. A notable initiative is the establishment of the CDC Consortium for COVID-19 Vaccine Clinical Trials by the African Union (AU) and Africa CDC [104]. This consortium aims to facilitate clinical trials for promising COVID-19 vaccine candidates and foster Africa-based vaccine manufacturing capacity, highlighting collaborative efforts among governments, UN agencies, and stakeholders. The commitment to local vaccine manufacturing was further reinforced through high-level summits, leading to the launch of the Partnerships for African Vaccine Manufacturing (PAVM) and the signing of crucial Memorandums of Understanding with organizations like the Coalition of Epidemic Preparedness Innovations [105]. The Partnerships for PAVM is a collaborative effort aimed at enhancing vaccine manufacturing capacity across the African continent. This initiative recognizes the critical need for Africa to strengthen its local vaccine production capabilities, particularly in light of the challenges posed by the COVID-19 pandemic. PAVM represents a strategic partnership between various stakeholders, including governments, international organizations, private sector entities, and research institutions. The initiative leverages collective expertise and resources to address key bottlenecks in vaccine manufacturing, such as technology transfer, regulatory harmonization, and infrastructure development. One of the primary objectives of PAVM is to facilitate technology transfer and knowledge sharing from established vaccine manufacturers to their African counterparts. This involves establishing partnerships with leading vaccine manufacturers worldwide to transfer manufacturing know-how, production processes, and quality control standards to African vaccine manufacturers. By bridging the technology gap, PAVM aims to empower African manufacturers to independently produce high-quality vaccines. Moreover, PAVM facilitates the establishment of vaccine production hubs and centers of excellence across Africa. These centers serve as focal points for vaccine research, development, and manufacturing, equipped with state-of-the-art facilities and staffed by skilled professionals. By consolidating resources and expertise in these hubs, PAVM accelerates the pace of vaccine production and innovation on the continent. In addition to technology transfer and capacity building, PAVM promotes regulatory harmonization to streamline the approval process for locally manufactured vaccines. This involves working closely with regulatory authorities in African countries to align regulatory standards, expedite approvals, and ensure compliance with international quality standards. By harmonizing regulatory frameworks, PAVM aims to create a conducive environment for vaccine manufacturing and facilitate the rapid deployment of life-saving vaccines. Furthermore, PAVM fosters collaboration between public and private sector entities to mobilize investment and resources for vaccine manufacturing projects. This includes facilitating partnerships between governments, pharmaceutical companies, development banks, and philanthropic organizations to fund infrastructure development, research initiatives, and training programs.

**Figure 2:**
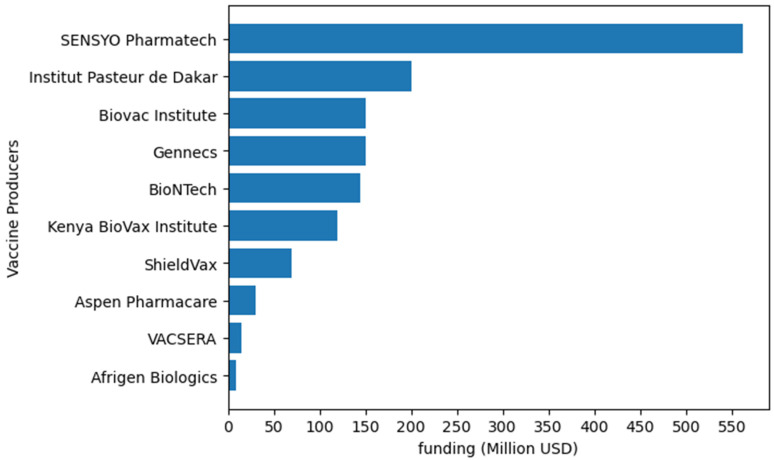
Examples of funding allocated to vaccine production by different African vaccine producers. Institut Pasteur de Dakar will invest up to USD 200 million [106]. Biovac will allocate up to USD 150 million [107]. BioNTech will utilize up to USD145 million from CEPI [108]. Afrigen Biologics will use USD 9 million [109]. Kenya BioVax Institute will invest up to USD 120 million [110]. ShieldVax plans to invest USD 70 million in vaccine production [111]. In 2022, VACSERA invested USD 15 million [112]. Gennecs will invest up to USD 150 million in vaccine production [113]. Aspen Pharmacare is set to invest USD 30 million in producing routine and outbreak vaccines [114]. SESYO Pharmatech intend to invest USD 562 million in vaccine manufacturing [115].

Investment in vaccine manufacturing (Figure 2) is crucial to ensure the sustainability of local production. Building such capacity requires substantial investment in production facilities, workforce training, regulatory capacity, and cold chain infrastructure. The AU has established the Pan PAVM initiative within the Africa CDC to drive its goal of producing 60% of the continent’s vaccine needs by 2040. Access to finance is one of the eight bold programs of PAVM’s Framework for Action, which is a deal preparation and financing facility. This facility makes provision for financing vaccine manufacturing initiatives as well as strengthening research and development and workforce development through Africa CDC’s network of collaborating partners.

By linking the strategic efforts of capacity building, international collaboration, and substantial financial investment, the African continent aims to not only enhance its vaccine manufacturing capabilities, but also ensure health security, promote economic development, and guarantee equitable access to vaccines across the continent.

In line with these efforts, key initiatives and centers have been proposed to advance vaccine manufacturing in Africa. The PAVM Initiative aims to promote sustainable human vaccine manufacturing capacity on the continent [116], while the Accelerating Vaccine Production in Africa initiative has emerged as a Center of Excellence, enhancing regional vaccine manufacturing capacity to ensure health security for the region [117]. Additionally, the EAC has contributed to this endeavor by establishing the EAC RCE-VIHSCM. This center focuses on providing high-quality training and disseminating best practices in vaccines, immunization, and health supply chain management, further reinforcing Africa’s commitment to local vaccine manufacturing and distribution.

In addition, under PAVM, the Africa CDC has recently engaged in initiatives to achieve the 2040 agenda of manufacturing 60% of vaccines on the continent by creating regional capability and capacity networks [118]. These networks aim to support the development and establishment of sustainable workforce development programs for the vaccine manufacturing ecosystem across the continent. These centers, located in up to five regions of Africa, will establish and operate sustainable training programs to build and maintain a skilled workforce for the vaccine manufacturing ecosystem both regionally and continent-wide.

The centers will increase the relevance of sustainably funded training and educational programs to meet evolving industry needs and incentivize R&D activities across Africa. This initiative is crucial for the Africa CDC and PAVM as it will energize efforts in this field and help achieve the goal of producing 60% of the vaccines administered on the continent locally by 2040. It will also facilitate coordinated efforts to align activities across regions, creating a unified manufacturing landscape [119].

## 11. Global Collaborations and Regional Agreements

**Figure 3:**
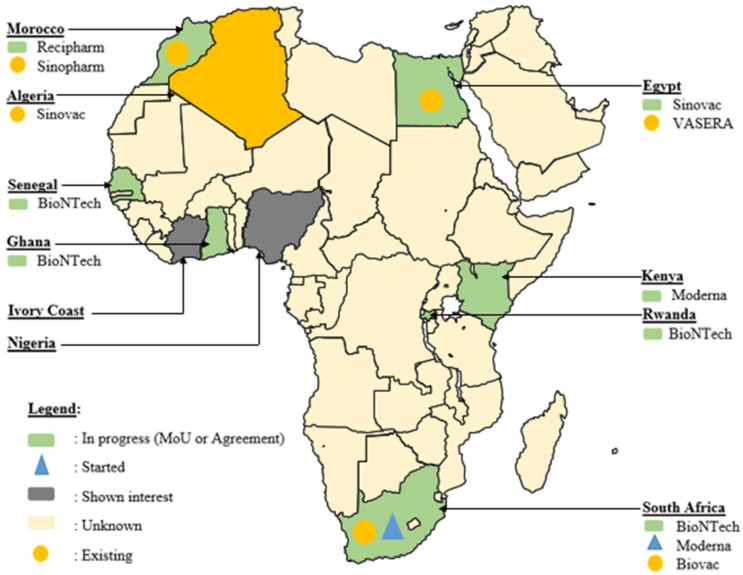
Geographical distribution of novel progress in vaccine development in Africa during the COVID-19 era (MoU: Memorandum of Understanding).

Africa’s commitment to overcoming vaccine challenges is evident in various agreements signed across the continent, many of which were initiated during and immediately after the COVID-19 pandemic era (Figure 3). In South Africa, significant strides have been made to bridge the vaccine production gap. The Biovac Institute in Cape Town and Aspen Pharmacare in Gqeberha, South Africa, have forged agreements for ‘fill and finish’ processes [120]. Additionally, during this period, Biovac Institute established a freezer farm facility for Pfizer to enhance the mRNA vaccine cold chain capacity, with plans to assess potential expansion at the Eastern Cape site to augment COVID-19 vaccine production. This collaborative effort, anticipated to have commenced in the second quarter of 2021, holds promise for bolstering local vaccine manufacturing capabilities [102,121].

Moreover, Aspen has expressed interest in undertaking the technology transfer and commercial manufacture of COVID-19 vaccines in collaboration with Johnson & Johnson, signaling a proactive approach to expanding the local vaccine manufacturing capacity [120]. It is noteworthy that while Aspen Pharmacare manufactured COVID-19 vaccines for Janssen, there was no uptake due to significantly decreased demand, leading to the destruction of the doses.

Amidst the challenges posed by the COVID-19 pandemic, the Biovac Institute has emerged as a key player in the WHO mRNA Technology Transfer Hub and Spoke model. Collaborating to scale up the validated commercial process of the COVID-19 mRNA vaccine developed by Afrigen Biologics, Biovac is instrumental in transferring this technology (via Afrigen Biologics) to spokes in Senegal (IPD), Nigeria (Biovaccines Nigeria), Tunisia (IPT), and Egypt (BioGeneric Pharma), bolstering their capacity to manufacture mRNA-based vaccines. Additionally, Biovac, in partnership with Pfizer-BioNTech, has expressed interest in producing mRNA COVID-19 vaccines. In East Africa, Rwanda has embarked on a significant project, in collaboration with BioNTech, the kENUP Foundation, and the International Finance Corporation, to produce COVID-19 and other vaccines using mRNA technology [122]. Furthermore, Uganda has initiated the construction of an mRNA vaccine manufacturing plant, with plans to produce vaccines for emerging diseases beyond COVID-19 in 2022 [123]. These agreements, forged amidst the pandemic, reflect a proactive stance in advancing local vaccine manufacturing capabilities to combat current and future health crises.

Even in Northern African countries such as Algeria, Egypt, and Morocco, active participation in the race for vaccine production was evident during the pandemic. For instance, Egypt’s VACSERA inked an agreement with China’s Sinovac Biotech Company to produce vaccines, while Minapharm and BioGeneric Pharma partnered with the Russian Direct Investment Fund to manufacture Sputnik vaccines [124]. Similarly, Algerian pharmaceutical company Saidal commenced bottling China’s Sinovac vaccine and secured an agreement to produce Russia’s Sputnik V. Additionally, Moroccan pharmaceutical company SOTHEMA entered agreements to produce Sinopharm and Sputnik V vaccines, underscoring the region’s commitment to local vaccine production and ensuring vaccine access for its population.

In West Africa, Senegal has shown interest in partnering with Belgium’s Univercells and the IPD to manufacture COVID-19 vaccines [102]. These collaborations and agreements exemplify the continent’s concerted efforts to strengthen its vaccine manufacturing capabilities and ensure equitable access to vaccines across Africa.

**Figure 4:**
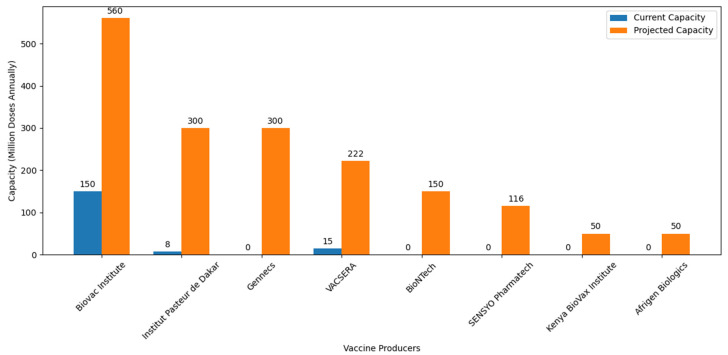
African vaccine production capacity: ambition and progress toward self-sufficiency. Information about the current and projected vaccine production capacities of various African vaccine producers were retrieved from different journals and press releases. BioNTech is expected to start producing 150 million doses annually across all of its facilities [125]. Afrigen Biologics is projected to produce 50 million doses annually [109]. VACSERA initially produced 15 million doses in its first batch and aims to produce 222 million doses annually [126]. Institut Pasteur de Dakar started with an initial production of 8 million doses [106] and plans to scale up to 300 million doses annually [127]. The Biovac Institute, which initially produced 150 million doses, is projected to increase its production to 560 million doses annually [128]. Kenya BioVax Institute estimating a production of USD 50 million doses annually [129]. Gennecs aims to produce 300 million vaccines per year [113]. SENSYO Pharmatech will manufacture 116 million vaccine units in 2024 [115].

Amid these collaborative efforts, it is crucial to acknowledge the recent introduction of the Vaccine Manufacturing Competency Framework. This framework serves as a strategic guide, delineating essential competencies required for navigating the intricate landscape of vaccine manufacturing. Its emphasis on comprehensive skill sets, from research and development to regulatory compliance and quality assurance, aligns seamlessly with Africa’s objectives in establishing self-sufficiency in vaccine production. Figure 4 illustrates significant planned increases in vaccine production capacities, with notable expansions at Institut Pasteur de Dakar (Senegal), Vacsera (Egypt), Biovac (South Africa), and new facilities such as Afrigen Biologics (South Africa) and BioNTech (Senegal, Rwanda, and South Africa).

The Competency Framework provides a timely resource for African nations, offering a roadmap to address challenges such as technology transfer barriers, regulatory gaps, and intellectual property concerns. As African countries strive to produce a significant proportion of their vaccines by 2040, the integration of the framework’s competencies into ongoing initiatives becomes imperative. This alignment ensures a more streamlined and effective approach, bolstering Africa’s position in the global vaccine manufacturing landscape.

Continued collaboration with international partners, as emphasized by the framework, can further enhance Africa’s capabilities and accelerate progress. By embracing the competencies outlined in this framework, African nations will be better equipped to build a skilled workforce and overcome hurdles in vaccine production, ultimately contributing to a healthier and more resilient continent.

These above agreements highlight the commitment and sustainable strategies of African institutions and the leadership of the AU in addressing long-standing vaccination challenges on the continent.

## 12. Challenges and Future Considerations

Addressing Africa’s knowledge gap in vaccine production is crucial for overcoming the existing challenges. Initiatives such as the Partnerships for PAVM play a pivotal role in this endeavor. By strengthening existing programs and offering short- and long-term courses in vaccinology, biomanufacturing, and related fields, Africa can upskill individuals, focusing on training to ensure practical proficiency.

Identifying personnel with backgrounds in life sciences is essential for spearheading vaccine design, development, and manufacturing. Establishing clear roadmaps to track their progress is equally vital for building a skilled workforce capable of contributing to Africa’s vaccine manufacturing objectives.

However, despite progress, several challenges persist. Intellectual property rights, licensing issues, and technology transfer barriers hinder advancement. Simplifying legislative frameworks and expediting approval processes for technology transfer could alleviate these obstacles. Additionally, sustaining vaccine production necessitates robust regulatory systems. Currently, many African countries lack comprehensive frameworks, impacting safety and efficacy assurance.

To build vaccine manufacturing capacity on the continent, a robust regulatory environment is of critical importance. The role of the African Medicines Agency (AMA) covers the broad themes of marketing authorization, inspection, market surveillance, safety and monitoring, oversight of clinical trials, and quality control. Its activities are complementary to and supportive of the functions of national regulatory authorities in the respective countries. Harmonizing legislation through developing common standards and regulations is crucial to ensuring a robust medical product regulatory environment on the continent [130]. In this context, the continent will have a common regulatory environment within which vaccine manufacturing capacity can be built and expanded.

Additionally, the AMA collaborates with RECs and the National Medicines Regulatory Authorities to identify and address substandard and falsified medical products, fostering a safer vaccine supply chain. The harmonization of legislation and the support for local pharmaceutical production under the PMPA present significant opportunities for enhancing the quality and safety of locally produced vaccines. This initiative aims to catalyze trade within the African Continental Free Trade Area and reduce reliance on imported vaccines, promoting sustainable healthcare development across the continent [130].

However, several challenges must be addressed to realize these goals. Some AU member states have been slow to join the AMA, which may hinder the uniform implementation of standards and regulatory practices. Strict mobilization efforts are essential to encourage full membership across Africa. Additionally, infrastructural and resource limitations could impede the local manufacturing capacity. Tackling these challenges requires coordinated efforts and investments in regulatory harmonization, capacity building, and infrastructure development. Furthermore, sensitizing and engaging non-profit organizations such as the International Vaccine Institute (IVI), GAVI, the World Health Organization (WHO), and other health funding institutions to support the AMA are crucial for its success. These collaborations can provide essential resources and expertise, bolstering the AMA’s efforts to ensure the availability of safe, effective, and locally produced vaccines across Africa. Encouraging countries to fully implement the AMA is imperative for improving access to quality medical products including vaccines.

In alignment with Africa’s quest for sustainable vaccine manufacturing, IVI plays a crucial role by collaborating with African countries to develop research and train individuals in vaccinology. The establishment of IVI’s Africa Regional Office in Rwanda signifies a significant step forward, providing on-the-ground support and leadership for vaccine-related initiatives across the continent. Additionally, IVI’s plans to open a Country and Project Office in Kenya, dedicated to its Advancing Vaccine End-to-End Capabilities initiative, further solidifies its commitment to Africa’s vaccine manufacturing goals.

AVEC Africa aims to accelerate the regional vaccine ecosystem by implementing end-to-end research and development projects tailored to local and regional needs. By fostering public–private partnerships and providing hands-on training, AVEC Africa aims to develop essential components for sustainable vaccine manufacturing in Africa.

These initiatives underscore IVI’s dedication to supporting Africa’s ambition of producing vaccines at a rate of 60% by 2040, thus contributing significantly to the continent’s journey toward self-reliance in vaccine production. Furthermore, Africa’s vaccine production must evolve beyond the current ‘fill and finish’ model to encompass end-to-end manufacturing including research and development to achieve sustainable and self-reliant vaccine production capabilities.

## 13. Conclusions

This review elucidates the critical role of vaccine education and human capacity development initiatives in Africa’s trajectory toward vaccine self-sufficiency. With steadfast commitment from governmental bodies, educational institutions, and collaborative partners, Africa’s future in vaccine production appears promising. Central to this endeavor is the establishment of comprehensive curricula and training programs in virology and vaccinology-related disciplines across tertiary institutions. The multifaceted training ecosystem advocated herein, comprising short-term skills courses, internships, and formal degree programs, coupled with robust investment in translational research infrastructure, is pivotal for fostering a skilled workforce, driving innovation, and re-training talent on the continent. Concurrently, prioritizing funding for higher education studies in vaccinology and facilitating investment opportunities in vaccine manufacturing are critical steps that African governments must undertake. Collaborative ventures with international organizations and private entities are integral to fortifying Africa’s capabilities in vaccine development and manufacturing. Addressing challenges such as intellectual property rights, technology transfer barriers, and regulatory discrepancies demands concerted action and strategic alignment. The establishment of the AMA stands as a testament to Africa’s commitment to harmonizing regulatory frameworks and enhancing access to quality medical products. Through sustained dedication and collective efforts, Africa is poised to emerge as a significant player in the global vaccine manufacturing landscape, safeguarding the health and well-being of its populace.

## Figures and Tables

**Table 1:** Vaccine-related programs according to African regions [up to December 2023].

SN	Region	Number of Countries	Number of Countries with Vaccines Related Programs		Number of Countries without Vaccines Related Programs		Number of Programs	
		n	n	%	n	%	n	%
1	Western Africa	16	5	31.3	11	68.8	10	62.5
2	Northern Africa	7	5	71.4	2	28.6	5	71.4
3	Eastern Africa	19	4	21.1	15	78.9	5	26.3
4	Southern Africa	5	2	40.0	3	60.0	6	120.0
5	Central Africa	9	4	44.4	5	55.6	5	55.6
	Total	56	20	35.7	36	64.3	31	55.4

**Table 2:** Status of vaccines for emerging and re-emerging diseases.

Disease	Cause	Most Affected Country in Last 5 Years	Available Vaccine	Mortality Rate	Refs.
Human African trypanosomiasis	Parasites of genus *Trypanosoma* and transmitted by infected tsetse flies.	70% of reported cases occurred in the Democratic Republic of the Congo, with an average of less than 1000 cases declared annually. It is still reported endemic in Central Africa.	No	Human African trypanosomiasis (sleeping sickness) was the first or second greatest cause of mortality in the affected communities, even ahead of HIV/AIDS.	[43,44,45]
HIV	The human immunodeficiency virus (HIV)	Eswatini	In progress	25%	[46]
Plague	Bacteria *Yersinia pestis*, a zoonotic bacteria, usually found in small mammals and their fleas.	Madagascar	Yes: A whole organism and subunits	30–100% if left untreated.	[47,48]
Hepatitis C	*Hepatitis C virus*	Egypt has the highest prevalence (17.5%) of HCV in the world	There is no effective vaccine against hepatitis C	5.3%	[49,50]
Malaria	Parasites that are transmitted to people through the bites of infected female Anopheles mosquitoes	Nigeria (31.3%)	Available	12%	[51,52]
Rift Valley fever	Mosquitoes and blood feeding flies	Egypt	An inactivated vaccine has been developed for human use, but it is not licensed and or commercially available.	<1%	[53,54,55]
Zika viruses	Parasites that are transmitted to people through the bites of infected Aedes species.	Cape Verde	Inactivated and DNA vaccine candidates under clinical trials.		[56,57,58]
Lassa fever	*Lassa virus* transmitted by rodent.	Benin, Ghana, Guinea, Liberia, Mali, Sierra Leone, Togo, and Nigeria.	Although promising candidates are being evaluated, as yet there are no approved vaccines or therapeutics for human use.	Diagnosis and prompt treatment are essential. The overall case-fatality rate is 1%. Among patients who are hospitalized with severe clinical presentation of Lassa fever, case-fatality is estimated at around 15%. Early supportive care with rehydration and symptomatic treatment improves survival.	[59,60]
Marburg virus infection or hemorrhagic fever	*Marburg virus*	Ghana, Guinea, and Uganda.	There are several candidates, but no approved vaccines or therapeutics for human use	90%	[61,62,63]
Ebola virus disease	*Ebola virus*	Democratic Republic of Congo (Zaire), Uganda (Sudan species), Guinea (Zaire species).	There are several candidates, but no approved vaccines or therapeutics for human use.	Zaire Ebola virus species (60–90%); Sudan Ebola virus species (40–60%).	[34,35,64]
Human monkey pox	Monkey pox virus	Nigeria	In progress at randomized phase 3 trial.	1–10%	[65,66,67]
Extensively drug-resistant tuberculosis (XDR TB)/multidrug-resistant tuberculosis (MDR TB)	*Mycobacterium tuberculosis*	Nigeria, South Africa Democratic Republic of Congo, Mozambique, Ethiopia, Angola, Kenya, United Republic of Tanzania.	Yes. Bacille Calmette-Guérin (BCG) is a vaccine for tuberculosis (TB) disease particularly in infants and small children. There is no current information on TB vaccines trials in Africa.	1.5 million deaths occurred worldwide; 3.3% being MDR-TB and XDR-TB strains in 2014 yet there is no accurate data estimating the current situation in Africa.	[68,69,70,71,72]
Cholera	*Vibrio cholerae*	Angola, Democratic Republic of the Congo, Mozambique, Ethiopia, Somalia, South Sudan, Sudan, and Zambia. Others are Nigeria, Somalia, Tanzania, and South Africa.	Yes. Oral cholera vaccines are available for the management of the disease.	160,930 deaths (52.6% of 2,548,227 estimated cases and 79.6% of 209,216 estimated deaths worldwide). Another estimates 1,411,453 cases and 53,632 deaths per year, respectively (50% of 2,836,669 estimated cases and 58.6% of 91,490 estimated deaths worldwide).	[73,74,75,76,77,78]
Respiratory syncytial virus (RSV)	Respiratory syncytial virus spread from person to person.	Globally but varies with season and weather conditions.	In Progress	1.0% for children younger than 1 year and 73.4% for adults aged 65 years or older.	[79,80]
Recently emerged COVID-19 pandemic	COVID-19 virus transmitted through contact (direct and through fomites), large droplets and aerosols.	China, USA, Italy, and Brazil in the first wave, while Brazil South Africa consistently reported more than 100,000 cases in 2022.	Yes	The global all-age rate of excess mortality due to the COVID-19 pandemic was 120.3 deaths (113.1–129.3) per 100,000 of the population.	[81,82,83]
Plague	*Yersinia pestis*	Madagascar, Democratic Republic of the Congo, Uganda, Malawi and Zambia. Sporadic cases have also occurred in Tanzania, Mozambique and Kenya.	The vaccine is given intramuscularly and comprises inactivated *Yersinia pestis* cells. For the purpose of maintaining long-term immunity, booster doses are necessary. Immunity is provided for around 6 to 12 months. Live attenuated vaccine: Given orally, this vaccine comprises weakened *Yersinia pestis* cells. It offers prolonged immunity and does not need booster doses.	There are three types of plague: Bubonic 50–60% without treatment, ˂5% with antibiotics. Septicemic 30–50% without treatment, ˂5% with antibiotics and pneumonic mortality rate almost 100% without intervention and ˂15% with antibiotics.	[84,85,86,87,88]
Chikungunya fever	*Chikungunya virus* (CHIKV)	Kenya, Tanzania, Madagascar, Comoros and Mozambique. Sporadic cases have also occurred in Uganda, Nigeria, and Senegal.	Currently no vaccine available for commercial use, however, some are currently in development and undergoing clinical trial (VLA1553).	Less than 0.1% (self-limited illness).	[89,90,91,92,93]

## Data Availability

The original contributions presented in the study are included in the Supplementary Materials, further inquiries can be directed to the corresponding author.

## Supplementary Materials

The following supporting information can be downloaded at: https://www.mdpi.com/article/10.3390/vaccines12070741/s1, Table S1: Institutions in Southern Africa with vaccine-related programmes. Table S2: Institutions in North Africa with vaccine-related programmes. Table S3: Institutions in Western Africa with vaccine-related programmes. Table S4: Institutions in Central Africa with vaccine-related programmes. Table S5: Institutions in Eastern Africa with vaccine-related programmes.
