# Supplementary material for: Advancing Vaccinology Capacity: Education and Efforts in Vaccine Development and Manufacturing across Africa
Source: Vaccines (Basel). 2024 Jul 3;12(7):741. doi: 10.3390/vaccines12070741 (PMC11281707; doi:10.3390/vaccines12070741)
Supplement: Supplementary file 1 [file vaccines-12-00741-s001.zip › vaccines-3024420-supplementary.pdf]

**Table S1:** Institutions in Southern Africa with vaccine-related programmes.

| Country | University/Institution | Programme name | Focus |
|---|---|---|---|
| **South Africa** | University of Witwatersrand | MSc (Med)Vaccinology | Develop scientific understanding of different aspects of vaccinology, and aid in the development of new vaccinesu |
| | Sefako Makgatho Health Science University | HCert Vaccinology | Provide knowledge about vaccine-preventable disease, vaccines, vaccination and immunisation |
| | University of Witwatersrand | African Advanced Vaccinology Course (Afro-ADVAC | Training in the field of vaccinology |
| | University of Cape Town | The Annual African Vaccinology Course | |
| | Sefako Makgatho Health Science University | Vaccinology Short courses | Introductory program to stay up-to-date in the field of vaccinology |
| **Zimbabwe** | University of Zimbabwe | MSc in Vaccinology | Develop and manufacture the badly needed vaccines for Africa |

**Table S2:** Institutions in North Africa with vaccine-related programmes.

| Country | Name of Institution | Programme | Focus |
|---|---|---|---|
| **Morocco** | National Institute of Hygiene | Master's degree program in Vaccinology | The fundamentals of vaccine development, manufacturing, and testing, as well as vaccination effectiveness and safety |
| **Tunisia** | University of Tunis El Manar | Faculty of Medicine, Master's degree program in Immunology and Vaccinology | The programme covers immunological concepts, vaccines' creation, and vaccinations' function in disease prevention. |
| **Algeria** | University of Algiers | Faculty of Medicine, Master's degree program in Immunology and Vaccinology | The program focuses on the principles of immunology and vaccine development, safety and efficacy. |
| **Morocco** | University of Hassan II | Faculty of Medicine and Pharmacy, Master's degree program in Biotechnology and Vaccinology | The program focuses on vaccination safety and effectiveness and the biotechnology and vaccine production fundamentals. |
| **Tunisia** | University of Monastir | Faculty of Pharmacy, Master's degree program in Vaccinology and Biotechnology | The program focuses on the principles of vaccine development, production, and evaluation, biotechnology and safety. |

**Table S3:** Institutions in Western Africa with vaccine-related programmes.

| Country | Name of Institution | Programme | Focus |
|---|---|---|---|
| **The Gambia** | The Medical Research Council Unit The Gambia at the London School of Hygiene & Tropical Medicine. | Short course since 2013 on Vaccinology in Africa: Masters level course held at the MRC Gambia at London School of Hygiene and Tropical Medicine. | Contribute to the evidence-based development and delivery of vaccines. |
| **Ghana** | 1. The University of Ghana.<br><br>2. The International Vaccine Institute (IVI) and the Kwame Nkrumah University of Science and Technology (KNUST).<br><br>3. West African Centre for Cell Biology of Infectious Pathogens (WACCBIP) | Short courses and R & D in vaccines production. | Effectiveness and clinical studies of different vaccines and increased campaign on immunisation. |
| **Mali** | University of Sciences, Techniques, and Technologies of Bamako (USTTB) | Short courses and training on vaccine/parasitology development, medical entomology, HIV/TB programmes. | Malaria epidemiology and vaccine studies;<br>Malaria immunological studies; Ecological studies on the malaria mosquito vectors;<br>Clinical studies on filariasis and clinical studies on HIV/TB, Ebola Virus Disease, Crimean Congo Hemorrhagic Fever Virus, and COVID-19. |
| **Nigeria** | 1. Pan African University Institute for Life and Earth Sciences- including Health and Agriculture (PAULESI), University of Ibadan (UI), Nigeria. | MSc. Veterinary Vaccine production & Quality Control option | Avian and Veterinary Medicine<br><br>Vaccine Production and Quality Control |

| | | | |
|---|---|---|---|
| | 2. National Veterinary Research Institute, Vom, Nigeria. | Advanced courses in vaccine research | Vaccines production and also conduct related research activities including veterinary sciences. |
| | 3. The Institute of Human Virology Nigeria. | Short courses on vaccines and immunisation with support from University of Maryland School of Medicine, Baltimore, USA, since 2004. | Training of healthcare workers, quality laboratory services, treatment adherence program that ensures access to medications and a support structure for adherence to prevent resistance to HIV, Tuberculosis and Malaria. |
| | 4. Usmanu Danfodiyo University, Sokoto. The Centre for Advanced Medical Research and Training (CAMRET). | Post-doctoral Research Fellow (Vaccinology and Molecular Virology) | Drug discovery, immunotherapeutics and vaccine development against infectious diseases, cancer, epigenetics and nutrigenomics. |
| | 5. Nigerian Institute for Medical Research (NIMR) Yaba, Lagos. | Professional courses on human virology and genomics. | Viral surveillance and Population Genomics<br>Development of Viral Diagnosis and Management Kits (NIMR Yellow Fever Virus RT-q PCR Kit; NIMR Lassa Fever Virus RT-q PCR Kit & Mpox RT-q PCR Kits) |
| **Senegal** | Ecole Inter-Etats des Sciences et Médicine Vétérinaires (EISMV) | Masters and Doctoral Degrees in Animal Health and Biotechnologies. | Provides the continent and the world with high-level multidisciplinary expertise in the field of animal production and health and public health. |
| | Institut Pasteur de Dakar (IPD) | International courses on arbovirology and on the surveillance and control of rabies | Vaccine production experience of over 80 years. The centre also provides high-quality services ranging from clinical diagnosis, human vaccination and food safety testing. |

**Table S4:** Institutions in Central Africa with vaccine-related programmes.

| Country | Name of Institution | Programme | Focus |
|---|---|---|---|
| **Central African Republic** | Institut Pasteur de Bangui | Master's degree program in Vaccinology and Biotechnology | The program focuses on vaccine development, production, and evaluation |
| **Cameroon** | University of Ngaoundere | Faculty of Science Master's degree program in Biomedical Sciences with a specialisation in Vaccinology | Vaccine research, development, and implementation. |
| **Democratic Republic of Congo** | University of Kinshasa | Faculty of Medicine, Master's degree program in Vaccinology and Immunology | Principles of vaccine development, administration, and their role in disease prevention |
| **Gabon** | Ecole Régionale de Santé Publique de Libreville | Diploma in Vaccinology | Introduction to the principles of vaccine development and administration |
| **Cameroon** | University of Buea | Faculty of Health Sciences, Master's degree program in Biomedical Sciences with a specialisation in Immunology and Vaccinology | Vaccine research, development and the principles of immunology |

**Table S5:** Institutions in Eastern Africa with vaccine-related programmes.

| Country | Name of Institution | Programme | Focus |
|---|---|---|---|
| **Rwanda** | University of Rwanda, EAC Centre of Excellence for vaccines, immunisation and health supply chain management | 1. Master of Science in Pharmaceutical Analysis and Quality Assurance | This program will ensure that the manufactured medication will provide the desired effect to the patient. It will also help graduates to be able to design, develop and manufacture vaccines and other biological products. |
| | | 2. Master of Science in Vaccinology | The focus of the programme is to provide knowledge and skills where academia, researchers, pharmaceutical industries, and policymakers will have a better understanding of the latest research and innovations, technical and commercial challenges involved in bringing new vaccine therapies to market. The overarching focus is to contribute to addressing community public health issues by producing graduates with high-level knowledge and skills in the design, production, and manufacture of vaccines. |

| | | | |
|---|---|---|---|
| **Uganda** | Uganda Virus Research Institute | Advanced Training on Vaccinology | Aimed to facilitate critical decision-making in vaccinology by providing participants with a comprehensive overview of the various aspects of vaccinology (immunology, vaccine development, clinical trials, regulatory processes, vaccine-specific issues, including new vaccines, vaccination strategies and policies, programme implementation, humanitarian emergencies, social, economic, political and ethical issues, financing, and communications) |
| **Kenya** | 1. Kenya AIDS Vaccine Initiative Institute of Clinical Research at University of Nairobi, Kenya | Vaccinology courses | Strengthening Clinicians, lab scientists, programme managers in vaccinology |
| | 2. Jomo Kenyatta University of Agriculture and Technology, Kenya | Masters in Infectious Diseases and Vaccinology | Teach and train Individuals with bachelors degree in biological science, medicine, pharmacy vaccines related skills |
| **Ethiopia** | Pan African University Institute of Life and Earth Science | Master's Degree Cert in Veterinary Medicine (vaccine production and management option | Focus on infectious diseases of animals and the development of vaccines |
